# Glycan Profiling in Small Extracellular Vesicles with a SERS Microfluidic Biosensor Identifies Early Malignant Development in Lung Cancer

**DOI:** 10.1002/advs.202401818

**Published:** 2024-06-17

**Authors:** Quan Zhou, Xueming Niu, Zhen Zhang, Kenneth O'Byrne, Arutha Kulasinghe, David Fielding, Andreas Möller, Alain Wuethrich, Richard J. Lobb, Matt Trau

**Affiliations:** ^1^ Centre for Personalised Nanomedicine Australian Institute for Bioengineering and Nanotechnology (AIBN) The University of Queensland Brisbane QLD 4072 Australia; ^2^ School of Biomedical Sciences Queensland University of Technology Brisbane QLD 4102 Australia; ^3^ Frazer Institute Faculty of Medicine The University of Queensland Brisbane QLD 4102 Australia; ^4^ Department of Thoracic Medicine Royal Brisbane and Women's Hospital Brisbane QLD 4029 Australia; ^5^ JC STEM Lab Li Ka Shing Institute of Health Sciences Department of Otorhinolaryngology Faculty of Medicine Chinese University of Hong Kong Shatin Hong Kong SAR 999077 China; ^6^ Tumour Microenvironment Laboratory QIMR Berghofer Medical Research Institute Brisbane QLD 4029 Australia; ^7^ School of Chemistry and Molecular Biosciences The University of Queensland Brisbane QLD 4072 Australia

**Keywords:** early diagnosis, extracellular vesicles, glycosylation, liquid biopsies, miniaturized biosensors, non‐small cell lung cancer, surface‐enhanced Raman spectroscopy

## Abstract

Glycosylation is the most common post‐translational modification of proteins and regulates a myriad of fundamental biological processes under normal, and pathological conditions. Altered protein glycosylation is linked to malignant transformation, showing distinct glycopatterns that are associated with cancer initiation and progression by regulating tumor proliferation, invasion, metastasis, and therapeutic resistance. The glycopatterns of small extracellular vesicles (sEVs) released by cancer cells are promising candidates for cancer monitoring since they exhibit glycopatterns similar to their cell‐of‐origin. However, the clinical application of sEV glycans is challenging due to the limitations of current analytical technologies in tracking the trace amounts of sEVs specifically derived from tumors in circulation. Herein, a sEV GLYcan PHenotype (EV‐GLYPH) assay that utilizes a microfluidic platform integrated with surface‐enhanced Raman scattering for multiplex profiling of sEV glycans in non‐small cell lung cancer is clinically validated. For the first time, the EV‐GLYPH assay effectively identifies distinct sEV glycan signatures between non‐transformed and malignantly transformed lung cells. In a clinical study evaluated on 40 patients, the EV‐GLYPH assay successfully differentiates patients with early‐stage malignant lung nodules from benign lung nodules. These results reveal the potential to profile sEV glycans for noninvasive diagnostics and prognostics, opening up promising avenues for clinical applications and understanding the role of sEV glycosylation in lung cancer.

## Graphical abstract

A surface‐enhanced Raman scattering‐based microfluidic assay is developed for profiling small extracellular vesicle glycosylation, to differentiate benign and early‐stage non‐small cell lung cancer patients, which is challenging by commonly used screening tools computed tomography and positron emission tomography.

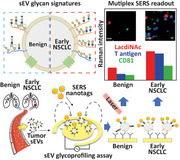

## 1 Introduction

Lung cancer has the highest rate of cancer mortality among both men and women, constituting almost 25% of all cancer‐related deaths.^[^ ^1^ ^]^ For the best chance of long‐term survival, early detection of lung cancer is of essential importance.^[^ ^2^ ^]^ Screening by low‐dose computed tomography (LDCT) has been shown to reduce mortality in lung cancer.^[^ ^3^ ^]^ However, there are limitations with this approach including high rates of false‐positives, substantial costs, and limited capability in differentiating malignant and benign lung nodules.^[^ ^4^, ^5^ ^]^ As a result, there is a significant unmet clinical need to develop accurate and minimally invasive tools to facilitate lung cancer screening that is capable of identifying early malignant lung nodules.

Liquid biopsies are minimally invasive approaches that can provide a view of the molecular landscape of the tumor microenvironment in bodily fluids (blood, urine, saliva, etc.).^[^ ^6^ ^]^ Recently, several liquid biopsy tests, such as CancerSEEK, PanSeer, and GRAIL, which are based on circulating tumor DNA (ctDNA) or circulating‐free DNA (cfDNA), have made significant advancements in the detection of multiple cancers.^[^ ^7^, ^8^, ^9^, ^10^ ^]^ However, these tests have very limited sensitivity for early‐stage cancer detection, due to extremely low abundance and variability in ctDNA levels among patients at the earliest stage of tumor development.^[^ ^7^, ^11^, ^12^ ^]^ Thus, alternative liquid biopsy approaches are required to enhance the sensitivity and specificity of early‐stage cancer detection.

Small extracellular vesicles (sEVs) have emerged as promising liquid biopsy biomarkers for cancer diagnosis.^[^ ^13^, ^14^ ^]^ sEVs are lipid‐bilayer membranous vesicles of 30–150 nm in diameter, released by almost all cell types and accumulate in circulating bodily fluids, such as blood, urine, and saliva.^[^ ^13^, ^15^, ^16^, ^17^ ^]^ Functioning as intercellular messengers, sEVs carry a diverse array of molecular contents, including nucleic acids, proteins, lipids, and glycans, which are carbohydrate modifications attached to proteins and lipids.^[^ ^18^ ^]^ Based on different glycan structures and linkages to the polypeptide backbone, protein glycosylation can be categorized into two main groups: *N*‐linked and *O*‐linked glycosylation.^[^ ^19^ ^]^ In cancer, aberrant alterations of glycosylation have been observed, including increased complexity of *N*‐glycans, truncated *O*‐glycans, and increased expression of sialylated and fucosylated terminals in both *N*‐linked and *O*‐linked glycans.^[^ ^20^ ^]^ Recently, several studies have identified characteristic features of sEV glycan profiles in melanoma, colon cancer, ovarian cancer, and prostate cancer.^[^ ^18^, ^20^, ^21^, ^22^ ^]^ In lung cancer, the role of sEV glycans and their potential to stratify benign and malignant lung nodules remained unexplored. As a result, the characterization of glycan profiles on sEVs may facilitate their translation to a noninvasive screening tool for lung cancer.

However, despite the thorough characterization of sEV proteins and nucleic acids, research on sEV glycans has been lacking.^[^ ^18^ ^]^ This lag can primarily be attributed to the complex structures of glycans and the lack of suitable analytical technologies. Although conventional methods such as mass spectrometry can detect sEV glycans, mass spectrometry requires specialized sample preparation, large sample amounts, advanced instrumentation, and complex data analysis.^[^ ^23^ ^]^ A simpler approach to glycan detection is a lectin microarray, which relies on carbohydrate‐binding proteins known as lectins.^[^ ^24^ ^]^ Fluorescently labeled sEVs are applied to lectins immobilized on an analytical chip and detected by their fluorescence signals.^[^ ^25^ ^]^ Emerging biosensor technologies have provided new momentum to sEV glycosylation research, for example, a microfluidic magnetoresistive biosensor was developed for the analysis of EV glycans in complex biofluids.^[^ ^26^, ^27^ ^]^ However, these glycan detection approaches are restricted to bulk glycan analysis of heterogeneous sEV populations and lack the ability to specifically identify tumor‐associated sEVs amidst the vast number of sEVs originating from normal, untransformed cells.^[^ ^26^ ^]^ This inherent limitation prevents the clinical translation of sEV glycosylation analysis.

To overcome these challenges, we developed a sEV GLYcan PHenotype (EV‐GLYPH) assay, which is based on a microfluidic platform integrated with surface‐enhanced Raman scattering (SERS) and lectin–glycan recognition for multiplex profiling of sEV glycans in non‐small cell lung cancer (NSCLC) patients. Previously we have shown that barcoding the protein content of single EVs can assess malignancy in lung nodules,^[^ ^28^ ^]^ while in this work we have developed a nanomixing platform technology to profile tumor‐associated sEV glycan patterns. To specifically analyze tumor‐associated glycans, sEVs were captured using anti‐mucin 1 (MUC1) antibody‐functionalized electrodes with the EV‐GLYPH assay. MUC1 is a glycoprotein biomarker that is associated with NSCLC.^[^ ^29^, ^30^ ^]^ Subsequently, two lectins, *Wisteria floribunda* agglutinin (WFA) and peanut (*Arachis hypogaea*) agglutinin (PNA) were conjugated with gold nanoparticles and Raman reporters as SERS nanotags, to recognize the glycans GalNAcβ1–4GlcNAc (LacdiNAc) and Gal(β1–3)GalNAc (T antigen) respectively on captured sEVs.^[^ ^13^, ^31^, ^32^ ^]^ LacdiNAc is a terminal group widely expressed in *N*‐ and *O*‐glycans in mammalian cells.^[^ ^33^ ^]^ Increased expression of LacdiNAc on *N*‐glycans has been identified on several glycoprotein biomarkers in prostate, ovarian, and colorectal cancers and is associated with proliferation, migration, invasion, and adhesion to extracellular matrices.^[^ ^33^, ^34^, ^35^, ^36^, ^37^, ^38^ ^]^ T antigen is an aberrantly truncated *O*‐glycan.^[^ ^39^ ^]^ While T antigen is masked on most healthy cells, it is over‐represented in malignant cells due to dysfunctional glycosyltransferases commonly found in multiple cancers.^[^ ^40^, ^41^, ^42^, ^43^, ^44^, ^45^, ^46^ ^]^ Using the EV‐GLYPH assay, we investigated the expression of LacdiNAc and T antigen on NSCLC‐associated sEVs, a previously unexplored biomarker profile in NSCLC. Leveraging the high sensitivity of our assay, we not only identify distinctive glycan patterns on NSCLC‐associated sEVs in an isogenic in vitro model but also successfully differentiate early‐stage NSCLC patients from subjects with benign lung conditions through sEV glycan analysis. As a result, our study represents the first report of SERS to analyze sEV glycosylation in lung cancer, paving the way for the use of sEV glycosylation profiles in the early diagnosis of NSCLC.

## 2 Results and Discussion

### 2.1 Principle of EV‐GLYPH Assay for Early‐Stage NSCLC Identification

**Figure 1:**
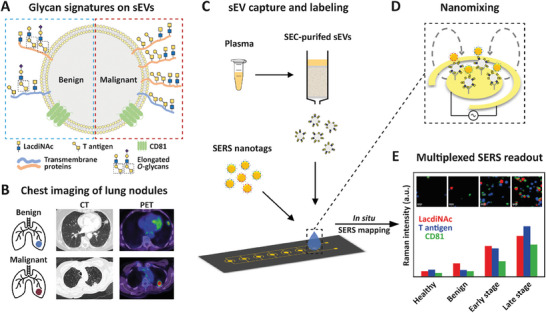
Principle of EV‐GLYPH assay for early‐stage NSCLC identification. A) Glycan signatures on sEVs derived from patients with benign and malignant lung diseases. B) CT and PET images of benign and malignant lung nodules. C) Working scheme of EV‐GLYPH assay. SEC‐purified sEVs from plasma are captured by anti‐MUC1 antibody immobilized on an electrode and subsequently labeled with SERS nanotags against LacdiNAc, T antigen, and CD81. D) A nanomixing fluid flow is generated on the electrode surface by the applied ac‐EHD field. E) With in situ SERS mapping, the average Raman intensities and false‐color SERS spectral images (insets) are established based on the characteristic Raman signals of three SERS nanotags (WFA‐MBA, 1075 cm^−1^, red; PNA‐TFMBA, 1375 cm^−1^, blue; anti‐CD81‐DTNB, 1335 cm^−1^, green), representing the expression of LacdiNAc, T antigen, and CD81, respectively.

We hypothesized that sEVs derived from NSCLC patients exhibit distinct glycan signatures (**Figure** **1**A), particularly increased expression of LacdiNAc and T antigen, compared to subjects with benign lung diseases. While imaging techniques, including computed tomography (CT) and positron emission tomography (PET), are commonly used tools for NSCLC detection,^[^ ^47^ ^]^ it is particularly challenging to differentiate benign and early‐stage malignant lung nodules as both may have similar features on CT and PET (Figure 1B), resulting in invasive biopsies and other tests being required for definite diagnosis of patients. To increase diagnostic accuracy and avoid unnecessary invasive biopsies, we therefore developed the EV‐GLYPH assay for profiling sEV glycosylation in NSCLC (Figure 1C). Plasma sEVs are purified via size exclusion chromatography (SEC), immobilized with an anti‐MUC1 antibody‐functionalized electrode surface, and subsequently labeled with a cocktail of SERS nanotags, which are gold nanoparticles conjugated with paired Raman reporters and lectins or antibody targeting LacdiNAc, T antigen and the canonical sEV marker protein CD81, respectively (Figure 1C). The characterization of gold nanoparticles and SERS nanotags is shown in Figure S1 (Supporting Information). The gold nanoparticles and SERS nanotags were spherical (Figure S1A, Supporting Information). The successful conjugation of antibodies and Raman reporters on the SERS nanotags was indicated by the increase in diameter (Figure S1B, Supporting Information) and hydrodynamic radius (Figure S1C, Supporting Information). By applying an alternating current–electrohydrodynamic (ac‐EHD) field onto a pair of asymmetric gold electrodes, a circulating fluid flow is generated at the electrode surface, which promotes the interaction of sEVs and SERS nanotags with the anti‐MUC1 antibody (Figure 1D).^[^ ^48^ ^]^ The fabrication process of gold electrodes is shown in Figure S2 (Supporting Information). Subsequent in situ SERS mapping generates false‐color spectral images based on the characteristic peaks of Raman reporters (Figure 1E). Signal intensities reflect the quantity of sEVs and the expression of the respective biomarkers on sEV surfaces, allowing the profiling of sEV glycosylation in NSCLC. We hypothesized that malignant patients possess a unique glycan signature of sEVs, thereby identifying early‐stage NSCLC.

### 2.2 Specificity of EV‐GLYPH Assay

**Figure 2:**
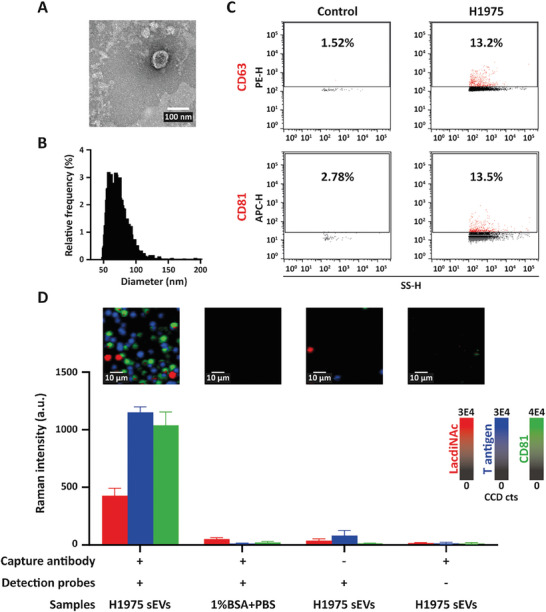
Specificity of EV‐GLYPH assay. A) TEM‐based evaluation, B) size distribution and quantification, and C) the expression of tetraspanins (CD63 and CD81) of H1975‐derived sEVs. Side scatter height (SS‐H) is proportional to particle size. D) Representative SERS false‐color spectral images and average Raman intensities of H1975‐derived sEVs, and control experiments including (++) 1% BSA+PBS, (−+) non‐targeted capture antibodies, and (+−) BSA on SERS nanotags. SERS false‐color spectral images and average Raman intensities were generated at 1075 cm^−1^ (LacdiNAc), 1375 cm^−1^ (T antigen), and 1335 cm^−1^ (CD81). Data are represented as mean ± standard error of three independent experiments. a.u., arbitrary units.

To investigate the specificity of the EV‐GLYPH assay in profiling glycosylation on NSCLC sEVs, we isolated sEVs from the NSCLC cell line H1975. We characterized the physical and biological properties of H1975‐derived sEVs, including morphology by transmission electron microscopy (TEM) (**Figure** **2**A), size distribution and abundance (Figure 2B), and expression of canonical tetraspanins CD63 and CD81 (Figure 2C).

After characterization, H1975 sEVs were utilized to investigate the specificity of our SERS‐based microfluidic assay. Control experiments included replacing sEVs with 1% (w/v) bovine serum albumin (BSA) in PBS, functionalizing electrode surfaces with non‐targeted antibodies, and functionalizing SERS nanotags with BSA. 1% BSA in PBS was used as the sample diluent due to lower nonspecific signals compared to PBS only (Figure S3, Supporting Information). As shown in Figure 2D, negligible signals were detected from the control experiments, while higher signals of SERS nanotags against LacdiNAc (12‐fold increase), T antigen (32‐fold increase), and CD81 (62‐fold increase) were observed on H1975 sEVs compared to control experiments, suggesting that NSCLC sEVs were specifically captured on the electrode and abundant LacdiNAc and T antigen were detected on NSCLC sEVs. Taken together, these data demonstrated that our EV‐GLYPH assay could specifically detect cancer‐associated glycosylation on NSCLC sEVs.

### 2.3 Sensitivity of EV‐GLYPH Assay

**Figure 3:**
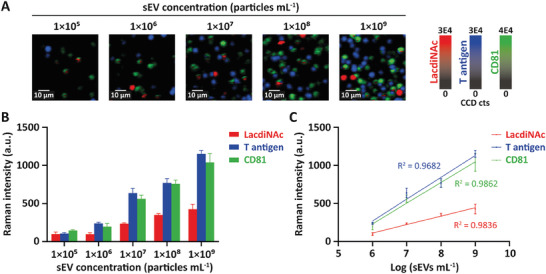
Sensitivity of EV‐GLYPH assay. A) Representative false‐color SERS spectral images and B) average Raman intensities at different sEV concentrations. False‐color SERS spectral images and average SERS intensities were generated at 1075 cm^−1^ (LacdiNAc), 1375 cm^−1^ (T antigen), and 1335 cm^−1^ (CD81). C) Linear regression curves between average Raman intensities and logarithmic sEV concentrations in the range of 1 × 10^6^ to 1 × 10^9^ particles mL^−1^. Data are represented as mean ± standard error of three independent experiments. a.u., arbitrary units.

To determine the assay sensitivity in the glycosylation profiling of NSCLC sEVs, we measured SERS intensities of purified H1975 sEVs at a concentration range of 1 × 10^5^ to 1 × 10^9^ particles mL^−1^. The frequency of signal dots and average signals increased with the elevating concentrations of purified H1975‐derived sEVs (**Figure** **3**A,B). Importantly, sEV concentrations between 1 × 10^6^ and 1 × 10^9^ particles mL^−1^ showed similar SERS signatures (Figure 3B), indicating that the lowest concentration of sEVs to produce a reliable SERS signal was 1 × 10^6^ particles mL^−1^. To determine the limit of detection (LOD) of the assay, we further conducted a linear regression analysis of individual biomarkers in the range of 1 × 10^6^–1 × 10^9^ particles mL^−1^ (Figure 3C). Raman intensities of each biomarker showed a linear relationship with increasing sEV concentrations, indicated by an *R* ^2^ value > 0.95. The LOD was calculated as 1 × 10^6^, 1 × 10^6^, and 1 × 10^5^ particles mL^−1^ for detecting LacdiNAc, T antigen, and CD81, respectively (Figure 3C). Based on these findings, the detection sensitivity for profiling glycosylation on sEVs derived from H1975 cells was determined to be 1 × 10^6^ particles mL^−1^.

### 2.4 Assessment of EV‐GLYPH Assay in an Isogenic In Vitro Model

After evaluation of the EV‐GLYPH assay performance in terms of specificity and sensitivity, we further investigated the capability of profiling sEV glycosylation in an isogenic cell line system. Untransformed human bronchial epithelial cell line (HBECs; 30KT) and an isogenic variant with oncogenic mutations commonly found in NSCLC, including p53 knockdown, KRAS^V12^ overexpression and LKB1 knockdown (30KT^p53/KRAS/LKB1^), were used.^[^ ^49^ ^]^ We hypothesized that such a model could reveal early changes in sEV glycopatterns that could indicate malignant transformation. Specifically, we expected that this would be indicated by the enrichment of LacdiNAc and T antigen on sEVs from transformed cells (30KT^p53/KRAS/LKB1^) compared to sEVs from non‐transformed cells (30KT).

**Figure 4:**
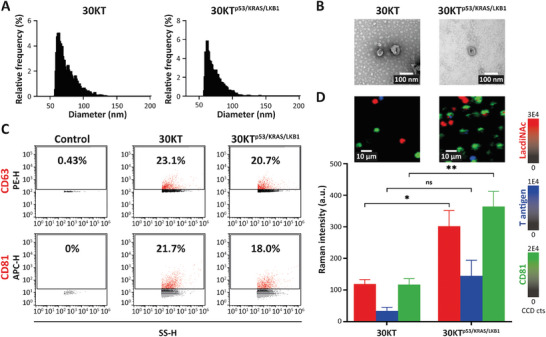
Glycan signatures of sEVs differentiate between non‐transformed and transformed HBECs. A) Size distribution and quantification, B) TEM‐based evaluation, and C) expression of tetraspanins (CD63 and CD81) of sEVs derived from 30KT and 30KT^p53/KRAS/LKB1^ cells. Side scatter height (SS‐H) is proportional to particle size. D) Representative SERS false‐color spectral images and average Raman intensities of sEVs derived from 30KT and 30KT^p53/KRAS/LKB1^ cells. SERS false‐color spectral images and average Raman intensities were generated at 1075 cm^−1^ (LacdiNAc), 1375 cm^−1^ (T antigen), and 1335 cm^−1^ (CD81). Data are represented as mean ± standard error of three independent experiments. a.u., arbitrary units. Two‐tailed unpaired *t*‐test was used to determine the significance. **p < 0.01; *p < 0.05; ns, not significant.

The size distribution, morphology, and expression of tetraspanins (CD63 and CD81) of sEVs purified from 30KT and 30KT^p53/KRAS/LKB1^ were characterized by TEM and nanoflow cytometry (**Figure** **4**A–C). Preliminary experiments using lectins for capturing and SERS nanotag against CD81 for detection indicated higher percentages of LacdiNAc^+^ and T antigen^+^ sEVs in transformed HBEC sEVs (Figure S4, Supporting Information). Subsequently, 30KT and 30KT^p53/KRAS/LKB1^‐derived sEVs were analyzed with our EV‐GLYPH assay. As shown in Figure 4D, elevated SERS signals of LacdiNAc, T antigen, and CD81 were observed on purified sEVs from transformed cells (30KT^p53/KRAS/LKB1^). These data showed that LacdiNAc, T antigen, and CD81 can potentially serve as diagnostic biomarkers for NSCLC.

### 2.5 Profiling sEV Glycans in Clinical Samples

**Figure 5:**
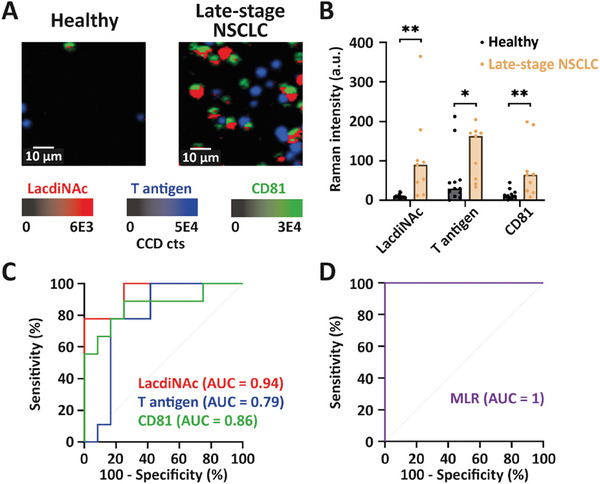
Analysis of sEV glycan signatures in healthy individuals and late‐stage NSCLC patients. A) Representative SERS false‐color spectral images and B) average Raman intensities of sEVs isolated from plasma of healthy individuals (*n* = 12) and late‐stage NSCLC patients (*n* = 9). SERS false‐color spectral images and average Raman intensities were generated at 1075 cm^−1^ (LacdiNAc), 1375 cm^−1^ (T antigen), and 1335 cm^−1^ (CD81). a.u., arbitrary units. Two‐tailed unpaired *t*‐test was used to determine the significance. **p < 0.01; *p < 0.05. C) ROC curves of individual biomarkers and D) ROC curve of MLR scores were conducted between healthy individuals and late‐stage NSCLC patients.

To determine the diagnostic capability of our EV‐GLYPH assay, we evaluated the accuracy of distinguishing sEVs isolated from the plasma of healthy individuals (*n* = 12) and late‐stage NSCLC patients (*n* = 9). Using an equal number of purified sEVs (2.5 × 10^8^ sEVs in a 50 µL sample), late‐stage NSCLC patients exhibited significant upregulation of LacdiNAc, T antigen, and the canonical sEV biomarker CD81 compared to healthy individuals (**Figure** **5**A,B). The median Raman intensities and ranges of LacdiNAc, T antigen, and CD81 were 8.49 (range: 4.08–21.54), 29.49 (range: 0–212.41), and 10.62 (range: 0.73–44.94) in healthy individuals, and 90.28 (range: 11.97–364.33), 163.29 (range: 33.47–205.13), and 64.91 (range: 8.24–199.19) in late‐stage NSCLC patients respectively. Receiver operating characteristic (ROC) curves were used to evaluate the diagnostic capability of EV‐GLYPH, with an area under the ROC curve (AUC) value of 1 reflecting a perfectly accurate diagnostic capability. LacdiNAc, T antigen, and CD81 exhibited significant diagnostic potential with AUC values of 0.94 (95% CI = 0.85 to 1.00), 0.79 (95% CI = 0.58 to 1.00), 0.86 (95% CI = 0.69 to 1.00), respectively (Figure 5C). Furthermore, we employed multiple logistic regression (MLR) analysis to combine the information from the three biomarkers. The ROC curve of MLR analysis demonstrated a superior diagnostic performance of our assay in distinguishing late‐stage NSCLC patients from healthy individuals, with an AUC value of 1 (95% CI = 1.00 to 1.00) (Figure 5D), with a negative predictive value (NPV) and positive predictive value (PPV) of 100%.

In a true cancer screening setting, many individuals may have inflammatory conditions or other benign diseases that could potentially increase the false‐positive rate. Given that it is particularly challenging to differentiate benign and early‐stage malignant lung nodules via common screening tools, for example, CT/PET imaging (Figure 1A),^[^ ^28^ ^]^ we obtained blood samples of 20 subjects with benign lung diseases and 20 early‐stage NSCLC patients for evaluation by the EV‐GLYPH assay. This sample size was predetermined based on the data obtained from the healthy and late‐stage NSCLC cohorts (Figure 5).^[^ ^50^ ^]^ That is, a sample size of 40 patients (i.e., 20 benign and 20 malignant) would be required to achieve a power of 90% with a 95% CI. The value for the power of EV‐GLYPH assay in healthy and late‐stage NSCLC cohort was 91.2%.

**Figure 6:**
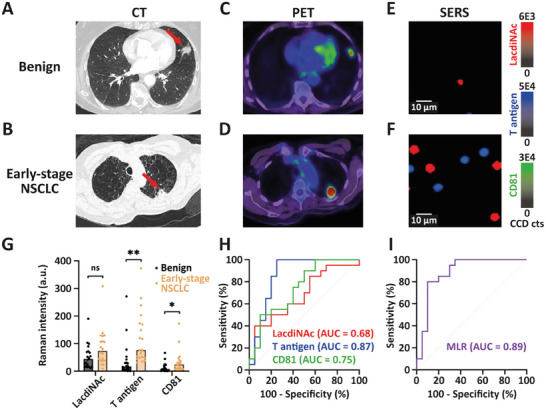
Implementation of EV‐GLYPH assay in a cancer screening setting. Representative A, B) CT, C, D) PET, and E, F) SERS false‐color spectral images of a benign subject and an early‐stage NSCLC patient. Red arrows indicated nodules in the patient's lungs. G) Average Raman intensities of sEVs isolated from plasma of subjects with benign lung diseases (*n* = 20) and early‐stage NSCLC patients (*n* = 20). SERS false‐color spectral images and average Raman intensities were generated at 1075 cm^−1^ (LacdiNAc), 1375 cm^−1^ (T antigen), and 1335 cm^−1^ (CD81). a.u., arbitrary units. Two‐tailed unpaired *t*‐test was used to determine the significance. **p < 0.01; *p < 0.05; ns, not significant. H) ROC curves of individual biomarkers and I) ROC curve of MLR scores were conducted between benign subjects and early‐stage NSCLC patients.

As shown in the representative CT images (**Figure** **6**A,B), the benign subject had a spiculated suspicious lesion in the lingula, while the early‐stage NSCLC patient had an air bronchogram (benign feature), demonstrating similar features are observed in benign and malignant nodules. The nodules in both patients were PET avid (Figure 6C,D), including the benign case, albeit with lower PET avidity. By contrast, in our EV‐GLYPH assay, the corresponding SERS images in Figure 6E,F show clear differences from the same quantity of sEVs (2.5 × 10^8^ sEVs in a 50 µL of sample), reflected by greatly increased signals from the early‐stage NSCLC patient compared to the patient with a benign nodule. In a statistically powered setting, we subsequently profiled a cohort of 20 benign and 20 early‐stage NSCLC patients. sEVs derived from early‐stage NSCLC patients exhibited elevated expressions of LacdiNAc, T antigen, and CD81 compared to benign subjects (Figure 6G). The median Raman intensities and ranges of LacdiNAc, T antigen, and CD81 were 43.97 (range: 10.67–190.41), 16.51 (range: 0–271.53), and 7.31 (range: 0–66.22) in benign subjects, and 73.64 (range: 9.16–308.71), 76.64 (range: 31.22–373.45), and 24.05 (range: 5.44–172.54) in early‐stage NSCLC patients respectively. The diagnostic performance of three biomarkers, assessed by ROC curves, demonstrated that T antigen displayed the best diagnostic classification, with an AUC value of 0.87 (95% CI = 0.74 to 0.99), while LacdiNAc and CD81 showed AUC values of 0.68 (95% CI = 0.51 to 0.85), and 0.75 (95% CI = 0.59 to 0.90), respectively (Figure 6H). By employing an MLR analysis to combine the three biomarkers, the diagnostic performance in classifying cases into benign or early‐stage NSCLC was further improved, with an AUC value of 0.89 (95% CI = 0.77 to 1.00) (Figure 6I). Cross‐validation by repeated random sub‐sampling was performed to assess the predictive performance of the EV‐GLYPH assay, showing an AUC of 0.86 (95% CI = 0.77 to 0.94). Furthermore, the NPV and PPV of the MLR were 84.21% and 80.95% respectively. These findings demonstrated that our EV‐GLYPH assay is capable of identifying early‐stage NSCLC patients by profiling sEV glycan signatures.

**Figure 7:**
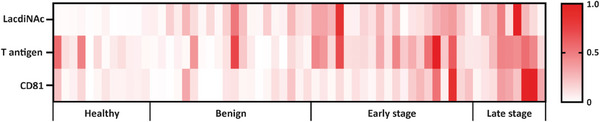
Heterogeneity of sEV glycan signatures among clinical samples. Heatmaps of LacdiNAc, T antigen, and CD81 on sEVs from healthy individuals (*n* = 12), subjects with benign lung diseases (*n* = 20), early‐stage NSCLC patients (*n* = 20), and late‐stage NSCLC patients (*n* = 9) were created based on normalized Raman intensities.

We then created heatmaps to visualize sEV glycan signatures of individuals from four cohorts based on normalized Raman intensities. As shown in **Figure** **7**, the expression levels of LacdiNAc, T antigen, and CD81 were upregulated in early‐ and late‐stage NSCLC patient cohorts, which is consistent with the representative SERS images (Figures 5A and 6E,F) and average Raman intensities (Figures 5B and 6G). Additionally, individuals within each cohort showed heterogeneous glycan signatures, demonstrating the intricate nature of glycosylation under both normal and pathological conditions, as well as the importance of utilizing multiple biomarkers for the early detection of NSCLC.

Our investigation into the feasibility of integrating sEV glycan signatures and nanotechnology for NSCLC screening lays the groundwork for future research aimed at expanding biomarker panels by harnessing the multiplex potential of SERS and broadening our assay's applicability in larger and more diverse patient cohorts for improved management of NSCLC. This multi‐marker approach shows promise in accurately discriminating individuals with malignant lung nodules from those with benign nodules or healthy subjects, indicating the potential clinical utility of our assay in early‐stage lung cancer detection. These findings highlight the potential of our EV‐GLYPH assay as a valuable tool in the clinical setting, contributing to improved patient outcomes and personalized treatment strategies. Future work on expanding this study into external cohorts in subsequent investigations will enable a more comprehensive evaluation of EV‐GLYPH's performance.

## 3 Conclusion

In conclusion, we developed a multiplex SERS‐based microfluidic assay named EV‐GLYPH for profiling glycans on NSCLC sEVs. This assay leverages the multiplexing capability of SERS due to its narrow spectral peak width, and enhanced assay specificity and sensitivity by ac‐EHD‐induced nanomixing effect.^[^ ^48^ ^]^ For the first time, we applied SERS to sEV glycosylation profile analysis for the early diagnosis of NSCLC. Our assay could detect 5 × 10^4^ sEVs in a 50 µL sample, which shows improved sensitivity compared to lectin microarray (1 × 10^7^–1 × 10^8^ sEVs, >100 µL) and mass spectrometry (1 × 10^10^ sEVs).^[^ ^51^, ^52^, ^53^ ^]^ This increased sensitivity of the EV‐GLYPH assay is due to the specific enrichment and detection of NSCLC‐associated sEVs. By successfully distinguishing between non‐transformed and transformed HBECs based on sEV glycan signatures, we have demonstrated the assay's ability to discern glycan alterations associated with carcinogenesis. In a clinical study, our assay revealed highly heterogeneous sEV glycan signatures and showed superior diagnostic performance in differentiating late‐stage NSCLC patients from healthy individuals, and early‐stage NSCLC patients from patients with benign lung diseases. These results highlight the significant potential of sEV glycans as valuable diagnostic biomarkers for detecting the early occurrence of NSCLC. By harnessing the unique glycan profiles of sEVs, the EV‐GLYPH assay opens up avenues for early intervention and improved patient outcomes in NSCLC.

## 4 Experimental Section

### Clinical Samples

All work done using human material was approved by the QIMR Berghofer Human Ethics Committee under P2180. The early‐stage NSCLC and benign samples were prospectively collected under the ACTRN12618001789257 trial. Here blood samples were taken from patients undergoing investigation of lung nodules prior to biopsy procedures. Malignant or benign outcomes were based on tissue biopsy results and clinical outcomes with radiologic stability or improvement over 12 months of CT follow‐up. Laboratory processing of blood samples was blinded to the clinical outcomes. Late‐stage NSCLC samples were collected at the Princess Alexandra Hospital with ethics approval obtained from the Metro South Health District Human Research Ethics Committee in accordance with the National Health and Medical Research Council guidelines (HREC/11/QPAH/331). Demographic data for subjects is indicated in Table S1 (Supporting Information). The comparison of age and gender in cohorts of healthy versus late‐stage (*p* < 0.0001) and benign versus early‐stage (*p* = 0.76) was performed using multiple linear regression analysis (Tables S2 and S3, Supporting Information).

### Cell Culture

Human NSCLC cell line H1975 was obtained from the American Type Culture Collection. Cells were cultured in RPMI 1640 medium (Gibco) supplemented with 10% fetal bovine serum (FBS, Gibco), 100 U mL^−1^ penicillin–streptomycin (Gibco), and 2 mм GlutaMAX, and kept in a humidified incubator in 5% CO_2_ at 37 °C. When cells reached 80% confluency, they were washed with 10 mм PBS and cultured in serum‐free media. Conditioned culture media (CCM) was collected after 48 h.

Isogenic immortalized normal human bronchial epithelial cells (HBEC30KT) transformed with p53 knockdown, KRAS^v12^ overexpression and LKB1 knockdown (30KT^p53/KRAS/LKB1^) were a gift from Dr. Jill Larsen.^[^ ^54^, ^55^ ^]^ HBECs were cultured in keratinocyte serum‐free medium (KSFM), supplemented with EGF (5 ng mL^−1^) and bovine pituitary extract (50 mg L^−1^), at 37 °C in 5% CO_2_. Bovine pituitary extract was depleted of bovine EVs through overnight centrifugation at 100 000 × *g*. When HBECs reached 20% confluency, they were incubated with EV‐depleted KSFM media. CCM was collected after 72 h.

### sEV Purification and Concentration

sEVs were purified and concentrated as previously described.^[^ ^56^, ^57^, ^58^ ^]^ CCM were centrifuged at 800 × *g* for 10 min at 4 °C and filtered by a 0.22 µm membrane to remove detached cells and cellular debris. Using a combination of SEC and ultrafiltration, 70 mL of CCM was transferred to a Centricon Plus‐70 100‐kDa centrifugal filter device (Merck) and concentrated to ≈250 µL by centrifugation at 3000 × *g* for 20 min at 4 °C. The concentrate was overlaid on qEV Legacy columns (IZON), followed by elution with 10 mм PBS. High particle/low protein fractions were pooled and concentrated in an Amicon Ultra‐2 10‐kDa centrifugal filter device (Merck).

Plasma was thawed rapidly and centrifuged at 4 °C at 1500 × *g* and 10 000 × *g* for 10 and 20 min respectively to remove debris and large vesicles. A total of 500 µL of clarified plasma was overlaid on qEV Legacy columns (IZON), followed by elution with 10 mм PBS. High particle/low protein fractions were pooled and concentrated in an Amicon Ultra‐4 10‐kDa centrifugal filter device (Merck).

### Nanoflow Cytometry

The concentrations and size distributions of purified sEVs were analyzed using nanoflow cytometry (NanoFCM Inc.) according to the manufacturer's protocol. 250 nm fluorescent silica microspheres (NanoFCM Inc.) and silica nanosphere cocktails 68–155 nm (NanoFCM Inc.) were used as references for the calculation of particle concentrations and size distributions, respectively. Based on the calibration curve constructed by running mixtures of silica nanospheres of various sizes, the SS‐H of every sEV could be converted into the corresponding particle size.

For tetraspanin profiling, ≈5 × 10^8^ particles of purified sEVs were incubated with either 0.25 µg of phycoerythrin (PE) anti‐CD63 (Thermo Fisher Scientific, clone H5C6), or 20 µL of allophycocyanin (APC) anti‐CD81 (BD Biosciences, clone JS‐81) antibodies for 1 h at room temperature. After incubation, free fluorescent dye‐conjugated antibodies were removed by Optima MAX‐XP tabletop ultracentrifuge (Beckman Coulter) at 110 000 × *g* for 25 min at 4 °C. The resulting pellet was then resuspended in 70 µL of 10 mм PBS for fluorescence analysis using nanoflow cytometry. The antibody‐only control was recorded for the same duration and used as the reference for gating.

To ensure robust analysis, ≈6000 particles were measured. Data were recorded and analyzed using NF Profession 1.0 Software and FlowJo version 10.8.1.

### Transmission Electron Microscopy (TEM)

For TEM analysis of sEVs, 2.5 µL of sEVs (1 × 10^11^ particles mL^−1^) were fixed with an equal volume of 2% glutaraldehyde for 30 min at room temperature. 5 µL of fixed sample was loaded on Formvar/carbon‐coated electron microscopic grids (Electron Microscopy Science) and incubated for 15 min. Excess liquid was removed by blotting. The grid was washed three times by brief contact with 100 µL of Milli‐Q water, followed by blotting to remove excess liquid. To contrast the sample, the grid was placed on 30 µL of 2% uranyl acetate (w/v) for 5 min and excess fluid was removed by blotting gently. Grids were left to air dry and observed using transmission electron microscopy (Hitachi HT7700) at 100 kV.

For TEM analysis of gold nanoparticles, particles were diluted in water and loaded on Formvar/carbon‐coated electron microscopic grids (Electron Microscopy Science). Grids were left to air dry and observed using transmission electron microscopy (Hitachi HT7700) at 100 kV.

### Scanning Electron Microscopy (SEM)

Gold nanoparticles were diluted in water and loaded on a silicon slice mounted on an SEM stub using carbon tabs. Samples were baked in a vacuum oven at 70 °C overnight and plasma cleaned before SEM analysis. SEM imaging was conducted using a JEOL JSM‐7800F FE‐SEM microscope with 10 kV voltage and a working distance of 10 mm.

### Differential Centrifugal Sedimentation

Differential centrifugal sedimentation was performed using a disc centrifuge (model DC24000 UHR, CPS instrument Inc.). The disc speed was set to 24000 rpm. 14.4 mL of sucrose gradient fluid comprising of 8%–24% w/v sucrose in water was loaded into the disc. The average density, refractive index, and viscosity of the sucrose gradient fluid were 1.069 g mL^−1^, 1.36, and 1.505 cps, respectively, according to the manufacturer's protocol. 0.5 mL of dodecane was added as a cap fluid. Polyvinyl chloride particles with a peak diameter of 0.483 µm and a density of 1.385 g mL^−1^ were used for calibration before each measurement. Calibration with standard particles allows the deconvolution of the optical signal into the particle size distribution.

### SERS Nanotag Synthesis

Gold nanoparticles were synthesized by citrate reduction of HAuCl_4_.^[^ ^59^ ^]^ 1 mL of 1% (w/v) trisodium citrate dehydrate (UNIVAR Solutions) was added to 100 mL of boiled 0.01% (w/v) HAuCl_4_. The mixture was then boiled for 20 min under magnetic stirring. 10 µL of 1 mм Raman reporters in ethanol (either 4‐mercaptobenzoic acid (MBA), 2,3,5,6‐tetrafluoro‐MBA (TFMBA), or 5,5′‐dithiobis (2‐nitrobenzoic acid) (DTNB)) and 2 µL of 1 mм dithiobis (succinimidyl propionate) (Thermo Fisher Scientific) in dimethyl sulfoxide were added into 1 mL of gold nanoparticles and incubated for 5 h at room temperature. The mixture was centrifuged at 5400 × *g* for 10 min and the supernatant was discarded. Particles were then resuspended in 200 µL of 0.1 mм PBS and incubated with 0.5 µg of WFA (Sigma–Aldrich, L1516), PNA (Sigma–Aldrich, L6135), or antibody against CD81 (Novus Biologicals, NB100‐65805) for 30 min at room temperature. The solution was then centrifuged at 600 × *g* for 10 min at 4 °C to remove free antibodies and resuspended in 200 µL of 0.1% (w/v) BSA (Life Technologies Australia Pty Ltd.) for gold nanoparticle surface blocking.

### Microchip Fabrication

The device with 28 asymmetric electrodes was fabricated with the standard photolithography according to a previous report.^[^ ^60^ ^]^ The electrode pattern was designed using Layout Editor L‐Edit V15 (Tanner Research) and written on 5‐inch soda lime chrome masks (Shenzhen Qingyi Precision Mask Making) using a direct write system µPG 101 (Heidelberg Instruments). A clean 4‐inch Boroflat wafer (Bonda Technology Pte Ltd) was then spin‐coated with a negative photoresist AZnLOF 2020 (Microchemicals GmbH) at 2500 rpm for 60 s. After a soft bake for 1 min at 110 °C, the wafer was UV‐exposed with the above‐prepared mask at a constant dose of 150 mJ cm^−1^ using a mask aligner (EVG 620, EV Group, St Florian am Inn), following a post‐exposure bake for 1 min at 110 °C and wafer development for 55 s using an AZ726 MIF Developer (Microchemicals GmbH). The gold electrodes were then created by deposition of 10 nm titanium and 200 nm gold using a Temescal FC‐2000 Deposition System (Ferrotec) and overnight lift‐off in Remover PG (Microchemicals GmbH). The wafer carrying the gold electrode structures was rinsed with isopropanol and dried under a flow of nitrogen.

To accommodate the liquid sample analysis, a 4 mm‐thick polydimethylsiloxane (PDMS) slab with microfluidic well structures was manually aligned to the electrodes of the device. The PDMS slab was prepared by curing activated silicon elastomer solution (Sylgard 184, Dow) for 2 h at 65 °C. The PDMS slab was then punched with 5 mm‐diameter wells and thermally bonded to the device overnight at 65 °C.

### Microchip Functionalization

20 µL of 5 mм dithiobis (succinimidyl propionate) in dimethyl sulfoxide was added to each well of the device and incubated for 40 min at room temperature. Wells were then washed once with ethanol and three times with 10 mм PBS. 20 µL of 10 µg mL^−1^ antibody against MUC1 (R&D Systems, MAB6298) was added into each well and incubated for 2 h at room temperature. 50 µL of 5% (w/v) BSA in 10 mм PBS was added into each well and incubated for 2 h at room temperature for blocking. Wells were then washed once with 1% (w/v) BSA in 10 mм PBS and ready for sEV capture.

### sEV Capturing and Labeling

2.5 × 10^8^ sEVs in a 50 µL of sample (i.e., purified sEVs diluted with 1% (w/v) BSA in 10 mм PBS) were added to each well where the alternating current–electrohydrodynamics (ac‐EHD) field (500 Hz and 800 mV) was applied to induce nano‐mixing for 45 min. The captured sEVs were then incubated with 25 µL of diluted SERS nanotags under the same ac‐EHD condition for 20 min. Between each incubation step, the wells were washed three times with 1% (w/v) BSA in 10 mм PBS to remove excess reagents.

### SERS Measurements

SERS mapping was performed with the WITec Alpha 300 R microspectrometer (Oxford Instruments) configured with a 633‐nm laser and a highly sensitive electron‐multiplying charge‐coupled device. The laser power of 4 mW and the system frequency were calibrated based on a peak at 520 cm^−1^ of the silicon wafer. SERS images were generated with a 50 ms integration time from each pixel at an area of 60 × 60 µm (60 pixels × 60 pixels) using a 20× microscope objective. Each sample was measured in triplicate.

### Statistical Analysis

The average SERS spectrum of each sample was calculated from the corresponding SERS image using Project FIVE software (Oxford Instruments). The height of characteristic peaks of each Raman reporter derived from the average SERS spectrum was used to plot the intensity bar graph (Figure S1D, Supporting Information). Autofluorescence background signals were then removed from raw SERS spectra using the Vancouver Raman Algorithm.^[^ ^61^ ^]^ Raman intensities at 1075 (MBA), 1375 (TFMBA), and 1335 (DTNB) cm^−1^ were calculated to represent the expression levels of LacdiNAc, T antigen, and CD81, respectively. Raman intensities of samples were blank corrected by subtracting the negative control for each run. The LOD was calculated according to the following equation: LOD (Raman intensity) = mean of blank + 3 × standard deviation of blank.^[^ ^62^ ^]^ The LOD (sEV concentration) was calculated by converting the LOD (Raman intensity) using the formula obtained from the calibration curve. Two‐tailed unpaired *t*‐test, MLR analysis, ROC curves, and multiple linear regression analysis were conducted by GraphPad Prism 9 (GraphPad Software Inc.). Differences with *p*‐values < 0.05 were considered significant (*p < 0.05, **p  <  0.01, ***p  <  0.001). MLR was performed by inputting Raman intensities into a multiple variables data table, followed by proceeding with multiple logistic regression analysis with the intercept and main effects model. Power analysis was performed using G*Power software (Ver. 3.1.9.6). Estimated sample size was calculated by selecting the *t*‐test (Means: Difference between two independent means) and A priori: Compute required sample size analysis. Input parameters were two tails, α error probability = 0.05, power = 0.90, and allocation ratio = 1. The effect size d was determined by means and standard deviations of Raman intensities of each biomarker in late‐stage NSCLC and healthy cohorts. The selected sample size was the maximum among the calculated sample sizes of three biomarkers. Cross‐validation was performed using Orange software (Ver. 3.35) by inputting Raman intensities of benign and early‐stage NSCLC cohort, choosing logistic regression as a learning algorithm, and selecting random sampling in the test and score module.^[^ ^63^ ^]^ The repeat train/test and training set size were set to 5 and 66% respectively. Multiple linear regression analysis was performed by inputting age and gender into a multiple variables data table, followed by proceeding with multiple linear regression analysis with the least squares method and the intercept and main effects model. Heatmaps were established based on the normalized Raman intensities, in which the smallest mean intensity was defined as 0 and the largest mean as 1. All figures were produced by Adobe Illustrator 2022 (Adobe Inc.).

## Conflict of Interest

The authors declare the following financial interests/personal relationships which may be considered as potential competing interests: Matt Trau has patent #US10156546B2 issued to University of Queensland. The authors declare no other competing interests.

## Supporting information

Supporting Information

## Data Availability

The data that support the findings of this study are available from the corresponding author upon reasonable request.
