# Supplementary material for: Glycan Profiling in Small Extracellular Vesicles with a SERS Microfluidic Biosensor Identifies Early Malignant Development in Lung Cancer
Source: Adv Sci (Weinh). 2024 Jun 17;11(33):2401818. doi: 10.1002/advs.202401818 (PMC11434045; doi:10.1002/advs.202401818)
Supplement: Supplementary file 1 — Supporting Information [file ADVS-11-2401818-s001.pdf]

ADVANCED SCIENCE
Open Access

Supporting Information

for *Adv. Sci.*, DOI 10.1002/advs.202401818

Glycan Profiling in Small Extracellular Vesicles with a SERS Microfluidic Biosensor Identifies Early Malignant Development in Lung Cancer

Quan Zhou, Xueming Niu, Zhen Zhang, Kenneth O'Byrne, Arutha Kulasinghe, David Fielding, Andreas Möller, Alain Wuethrich, Richard J. Lobb* and Matt Trau**

Supporting Information

Glycan profiling in small extracellular vesicles with a SERS microfluidic biosensor identifies early malignant development in lung cancer

Quan Zhou, Xueming Niu, Zhen Zhang, Kenneth O'Byrne, Arutha Kulasinghe, David Fielding, Andreas Möller, Alain Wuethrich, Richard J. Lobb*, Matt Trau**

Q. Zhou, X. Niu, Dr. Z. Zhang, Dr. A. Wuethrich, Dr. R. J. Lobb, Prof. M. Trau
Centre for Personalized Nanomedicine, Australian Institute for Bioengineering and Nanotechnology (AIBN), The University of Queensland, QLD 4072, Australia.
Email: a.wuethrich@uq.edu.au; richard.lobb@uq.edu.au; m.trau@uq.edu.au

Prof. K. O'Byrne
School of Biomedical Sciences, Queensland University of Technology, QLD 4102, Australia.

Dr. A. Kulasinghe
Frazer Institute, Faculty of Medicine, The University of Queensland, QLD 4102, Australia.

A/Prof. D. Fielding
Department of Thoracic Medicine, Royal Brisbane and Women's Hospital, QLD 4029, Australia.

Prof. A. Möller
JC STEM Lab, Li Ka Shing Institute of Health Sciences, Department of Otorhinolaryngology, Faculty of Medicine, Chinese University of Hong Kong, Shatin, Hong Kong SAR.

Prof. A. Möller
Tumour Microenvironment Laboratory, QIMR Berghofer Medical Research Institute, QLD 4029, Australia.

Prof. M. Trau
School of Chemistry and Molecular Biosciences, The University of Queensland, QLD 4072, Australia.

Figure S1. Characterization of gold nanoparticles and SERS nanotags.

Figure S2. Fabrication process of the device.

Figure S3. Comparison of sample diluents.

Figure S4. Comparison of lectins to capture HBEC-derived sEVs.

Table S1. Demographic data for subjects.

Table S2. Comparison of age and gender between health individuals and late-stage NSCLC patients.

Table S3. Comparison of age and gender between subjects with benign lung diseases and early-stage NSCLC patients.

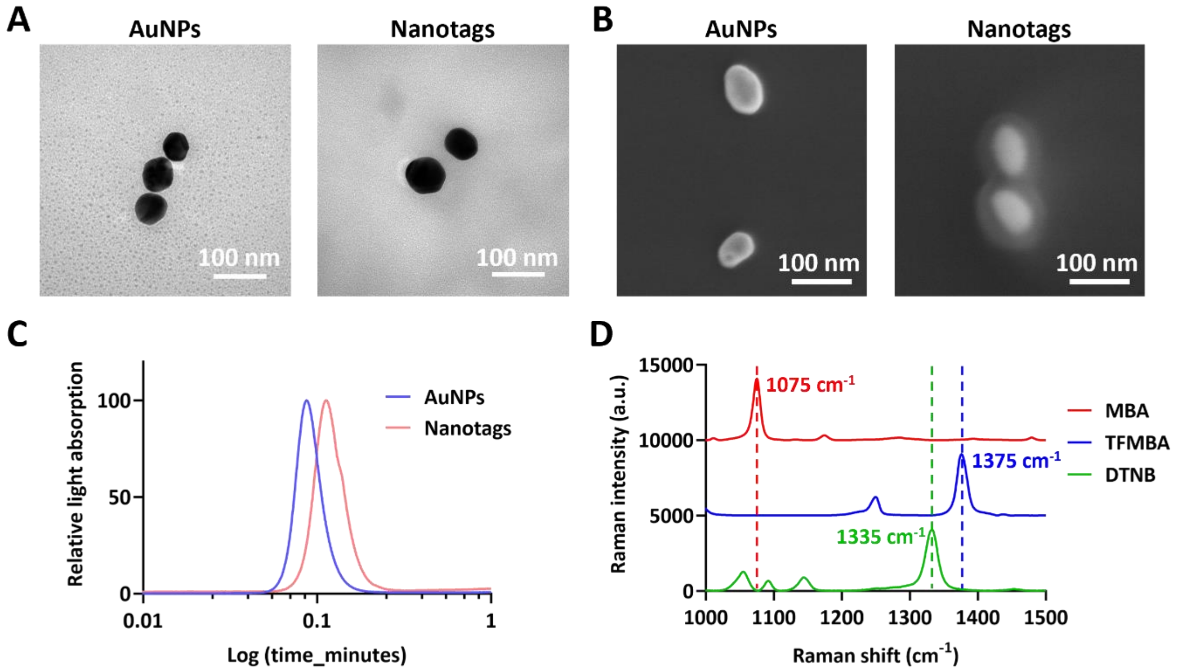

Figure S1. Characterization of gold nanoparticles and SERS nanotags. Gold nanoparticles (AuNPs) and SERS nanotags are characterized by (**A**) TEM, (**B**) SEM and (**C**) differential centrifugal sedimentation. For differential centrifugal sedimentation analysis, analytical time is proportional to particle size. (**D**) Raman spectra of SERS nanotags (MBA, 1075 cm^{-1}, red; TFMBA, 1375 cm^{-1}, blue; DTNB, 1335 cm^{-1}, green). a.u., arbitrary units.

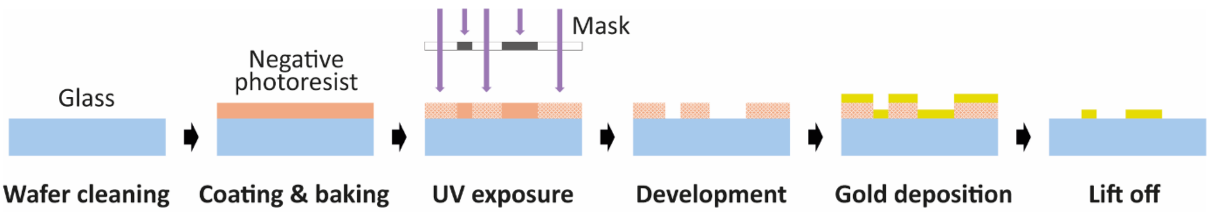

Figure S2. Fabrication process of the device. A clean glass wafer is spin-coated with a negative photoresist and baked. Subsequently, the wafer is UV-exposed using a soda lime chrome mask with designed electrode patterns, following a post-exposure bake. The wafer is developed, and gold electrodes are created by deposition of titanium and gold, following the lift-off process.

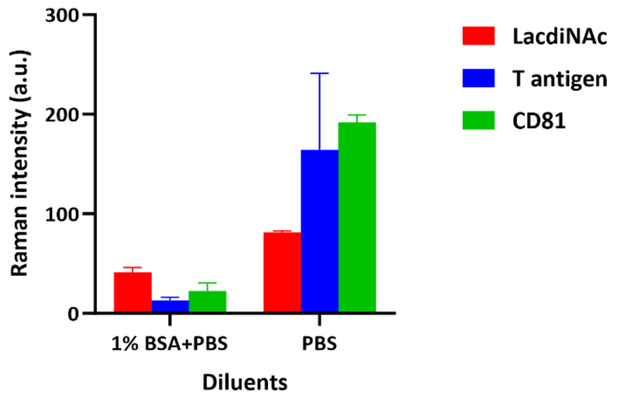

Figure S3. Comparison of sample diluents. Experiments were performed with 1% BSA in PBS or PBS, captured by antibodies against MUC1 and detected by SERS nanotags against LacdiNAc, T antigen and CD81. Raman intensities were generated at 1075 cm^{-1} (LacdiNAc), 1375 cm^{-1} (T antigen), and 1335 cm^{-1} (CD81). Data are represented as mean ± standard error of three independent experiments. a.u., arbitrary units.

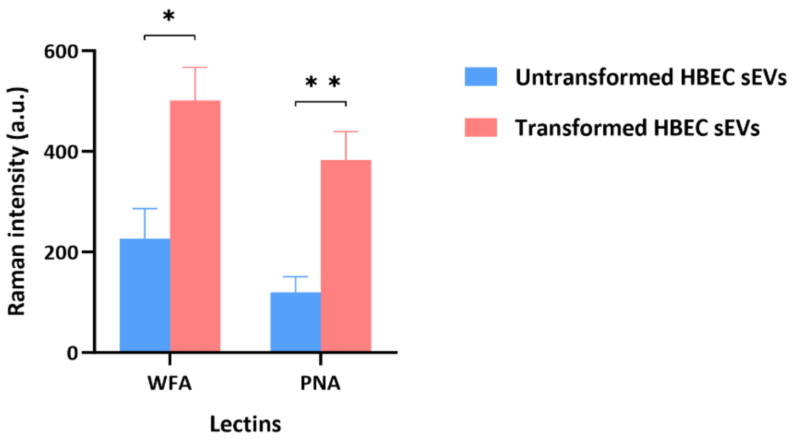

Figure S4. Comparison of lectins to capture HBEC-derived sEVs. sEVs derived from untransformed HBECs and malignantly transformed HBECs were captured by WFA or PNA individually and detected with SERS nanotag against canonical sEV marker CD81. Data are represented as mean ± standard error of three independent experiments. a.u., arbitrary units. Two-tailed unpaired t-test was used to determine the significance. ** $P < 0.01$; * $P < 0.05$.

Table S1. The demographic data of NSCLC patients (n = 29), subjects with benign lung diseases (n = 20), and healthy individuals (n = 12).

Patient ID	Gender	Age	Stage
P1	F	60	IV
P2	M	34	IV
P3	M	71	IV

P4	M	47	IV
P5	M	75	IV
P6	M	64	IV
P7	M	67	IV
P8	M	59	IV
P9	M	57	IV
P10	F	66	I
P11	F	75	I
P12	M	74	I
P13	F	71	I
P14	M	71	I
P15	F	67	I
P16	M	53	I
P17	M	71	I
P18	F	83	I
P19	F	61	I
P20	F	72	I
P21	M	51	I
P22	F	75	I
P23	M	67	I
P24	M	61	I
P25	M	48	I
P26	F	55	I
P27	F	75	I
P28	F	69	I
P29	M	71	I
B1	M	71	N/A
B2	M	72	N/A
B3	F	67	N/A
B4	F	55	N/A
B5	M	71	N/A
B6	M	70	N/A
B7	F	58	N/A
B8	F	47	N/A
B9	F	72	N/A
B10	F	78	N/A
B11	F	65	N/A
B12	M	76	N/A
B13	F	83	N/A
B14	F	67	N/A

B15	M	57	N/A
B16	F	79	N/A
B17	F	56	N/A
B18	M	70	N/A
B19	F	51	N/A
B20	F	46	N/A
H1	F	27	N/A
H2	M	24	N/A
H3	F	28	N/A
H4	M	29	N/A
H5	M	25	N/A
H6	M	27	N/A
H7	M	30	N/A
H8	F	27	N/A
H9	F	29	N/A
H10	F	29	N/A
H11	M	29	N/A
H12	F	28	N/A

Table S2. Comparison of age and gender between health individuals (n = 12) and late-stage NSCLC patients (n = 9) using multiple linear regression analysis.

		Healthy individuals	Late-stage NSCLC patients
Gender	Female	6	1
	Male	6	8
Age	Mean ± SD	27.7 ± 1.7	59.3 ± 11.8
P value		< 0.0001	

Table S3. Comparison of age and gender between subjects with benign lung diseases (n = 20) and early-stage NSCLC patients (n = 20) using multiple linear regression analysis.

		Subjects with benign lung diseases	Early-stage NSCLC patients
Gender	Female	13	11
	Male	7	9
Age	Mean ± SD	65.6 ± 10.5	66.8 ± 9.0
P value		0.76	
